# Motion cues tune social influence in shoaling fish

**DOI:** 10.1038/s41598-018-27807-1

**Published:** 2018-06-28

**Authors:** Bertrand Lemasson, Colby Tanner, Christa Woodley, Tammy Threadgill, Shea Qarqish, David Smith

**Affiliations:** 1 Environmental Lab, U.S. Army Engineer Research and Development Center (ERDC), Newport, Oregon USA; 2 Misericordia University, Dallas, PA USA; 3 Environmental Laboratory, ERDC, Vicksburg, MS USA; 4 College of Osteopathic Medicine, William Carey University, Hattiesburg, MS USA

## Abstract

Social interactions have important consequences for individual fitness. Collective actions, however, are notoriously context-dependent and identifying how animals rapidly weigh the actions of others despite environmental uncertainty remains a fundamental challenge in biology. By exposing zebrafish (*Danio rerio*) to virtual fish silhouettes in a maze we isolated how the relative strength of a visual feature guides individual directional decisions and, subsequently, tunes social influence. We varied the relative speed and coherency with which a portion of silhouettes adopted a direction (leader/distractor ratio) and established that solitary zebrafish display a robust optomotor response to follow leader silhouettes that moved much faster than their distractors, regardless of stimulus coherency. Although recruitment time decreased as a power law of zebrafish group size, individual decision times retained a speed-accuracy trade-off, suggesting a benefit to smaller group sizes in collective decision-making. Directional accuracy improved regardless of group size in the presence of the faster moving leader silhouettes, but without these stimuli zebrafish directional decisions followed a democratic majority rule. Our results show that a large difference in movement speeds can guide directional decisions within groups, thereby providing individuals with a rapid and adaptive means of evaluating social information in the face of uncertainty.

## Introduction

Social information is a central component that shapes collective behaviors across contexts and can enhance individual fitness^1–3^. While animals have evolved numerous cognitive mechanisms to help them distill information from their surroundings^4^, a noisy environment or the need for haste can create uncertainty. Social animals can reduce their uncertainty^5,6^ and responds to threats faster^7–9^ by pooling their information, although how they do so has important ramifications regarding the results of their actions^10–13^. Indiscriminate copying of others is not adaptive and animals are likely to have either evolved signals^14^, or strategies^15^ to help them selectively sample social information. The difficulty group members face is knowing who to attend to, as most social information is shared inadvertently through discrete behavioral cues that are ephemeral and can be ambiguous^3^. Consequently, questions remain as to how individuals living in groups, particularly those on the move, can leverage their sensory mechanisms to reduce any ambiguities associated with their social interactions.

Identifying general sensory mechanisms that influence collective animal behaviors, and translate across taxa, has been challenging. Correlation and multivariate analyses of animal groups in motion have revealed structural patterns in the speed and turning behaviors of individuals^16,17^, hierarchical associations^18^, and the capacity for extensive self-similarities across individuals regardless of group size (i.e., scale-free associations)^19^. While these approaches have provided insights into what are essentially dynamic, interacting systems, extracting causal interactions from systems plagued by collinearities remains problematic. It is therefore important to balance these alternative approaches with hypothesis driven, controlled experiments^20^. In particular, there remains the need to link how sensory information influences the individual’s reliance on social cues. For instance, many of the benefits of living in groups have often been observed to improve as group size increases^9,21,22^, yet theory also suggests that these benefits are context-dependent and that individuals can sometimes be better off in smaller groups^23^, or alone^23^ based on how environmental uncertainty influences their decision-making.

Vision is a crucial sensory modality during locomotion in most animals, from flies to humans, that enables individuals to filter out redundant information^4^, stabilize movements^24,25^, and execute some of the fastest neuromotor responses recorded in the animal kingdom^26^. Visual information is also an important sensory modality in shaping social interactions^12,27^ and may therefore provide clues as to how social information spreads quickly through groups regardless of context. Modeling studies demonstrate that interactions governed by simple visual properties, such as gaze limits^28,29^, occlusion^12^, or selective attention^11,13^ provide a better explanation for the emerging geometries and information cascades found in groups than do models that merely constrain interactions using alternative means (e.g., a distance or topological interaction limit).

Fish continue to provide a promising avenue to explore the role of visual cues on dynamical social interactions. Zebrafish, *Danio rerio*, form social groups characterized by elastic memberships called shoals, whose collective coherency ranges from disorganized to highly coordinated^30^. The visual and motor-control systems of zebrafish have also been well studied. Zebrafish have an extensive field of view^31^ and display an optomotor response to visual stimuli within days of hatching, whereby individuals can be induced to either follow, or flee from, visual images^32,33^. The optomotor response is a reflexive behavior displayed by many taxa as a means of stabilizing retinal image motion by adjusting movement speeds to account for patterns in their optic flow. However, little is known of how such visually driven reflexive responses vary with the coherency of neighbor activity, or how such responses integrate with social feedbacks to influence the speed and accuracy of decisions made within groups. Here we capitalize on the above characteristics to test the hypothesis that a salient visual cue, motion, can guide an individual’s attention within a group, thereby influencing their directional decisions and, ultimately, tuning the strength of any social feedbacks^11^. We begin by establishing the importance of relative speed in eliciting a robust reflexive response from individuals, despite varying levels of visual coherency that are common in fish shoals. We then demonstrate how a large change in the speed of visual cues provides a mechanism to tune the individual’s reliance on social information within groups, and that social interactions, while accelerating decision-making events, do not necessarily help individuals circumvent their speed-accuracy trade-off.

## Results

An individual’s ability to detect motion in its visual field typically improves with the directional coherency or relative speed of the cues^34–38^. If the relative speed of a set of visible motion cues, Δ*v*, influences an individual’s overt attention within a group, then we may expect an interaction between Δ*v* and the coherency of those cues, *C* on a subject’s directional decisions. We may also expect that the relative strength of a set of visual cues (coherency and/or speed) will subsequently influence how individuals respond to social information (neighbor decisions). To test these predictions, we first ran solitary zebrafish through a maze in which we controlled the speed and coherency of virtual silhouettes designed to mimic neighboring fish. Initially omitting other conspecifics provided a baseline response to our two visual factors of interest, while also controlling for confounding collinearities from group members (phenotypic variability, pair-wise associations, or physical forces from crowding). Based on our findings we then fixed the coherency of the visual stimuli and included group size as a factor. These conditions enabled us to isolate how social feedbacks from real neighbors and a large difference in the relative speed of the visual cues combined to influence decision-making in animal groups on the move.

### Sensory maze

**Figure 1:**
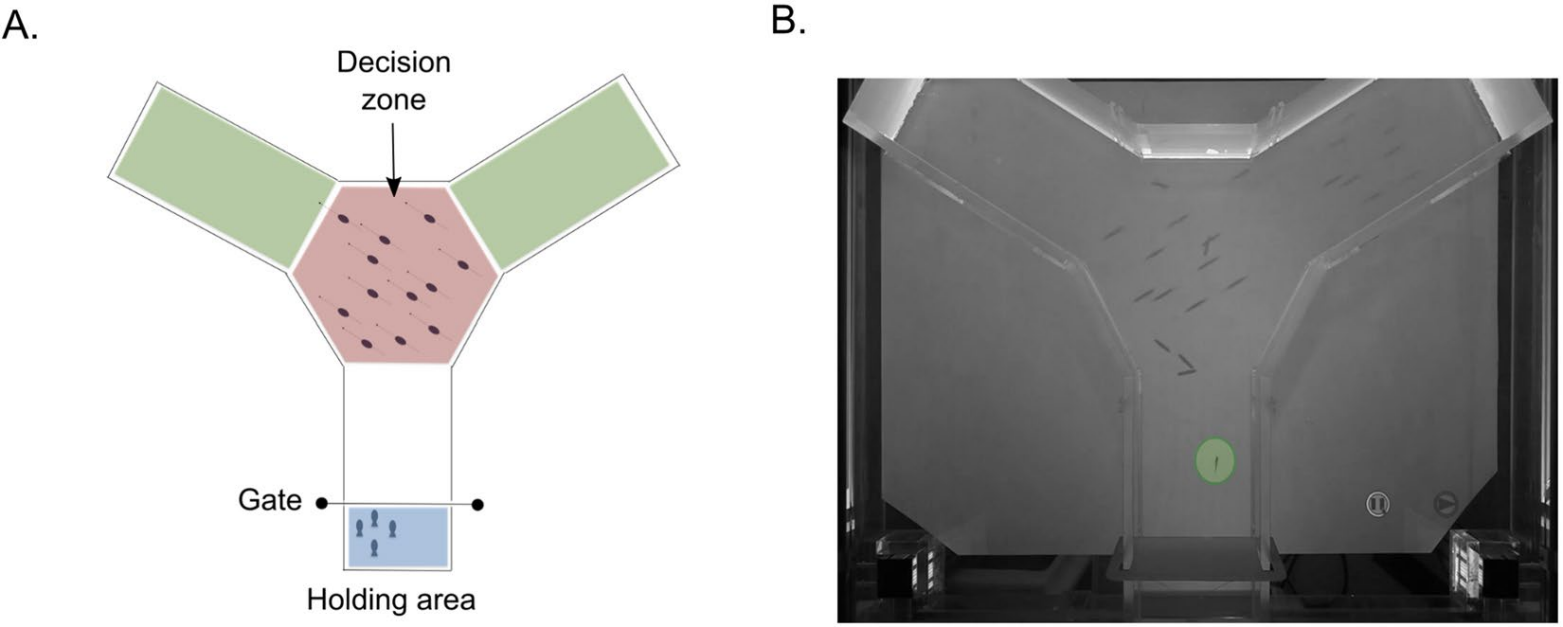
Experimental domain. Panel (A) illustrates the relative locations of the holding area (blue), decision zone in which the virtual fish silhouettes were projected (red), and the destination arms (green). Panel (B) shows a grayscale image of the domain in which a zebrafish (orange circle) makes its way up from the holding area towards the decision zone where the virtual silhouettes are projected. The virtual shoal’s coherency here is *C* = 0.67, with leader silhouettes moving towards the right arm.

The use of virtual tools has steadily increased in both application and sophistication to study complex behaviors in animals and humans^39–42^, providing novel ways to isolate confounding factors in controlled experiments. Here we used a simple 2D system to project visual stimuli onto the bottom of a Y-maze’s decision zone from below, thereby mimicking the silhouettes of conspecifics swimming beneath the subjects in the water column (Fig. 1). The silhouettes were the same size and shape as our average subject and served as virtual ‘leaders’ by moving directly towards a randomly selected arm in the maze using ballistic motion at a baseline speed equivalent to 1 body length per second for our subjects (≈3.5 cm ⋅ s^−1^). We tuned the coherency of these stimuli (*C*:0.00, 0.33, 0.66, 1.00) by including noise in the form of distractors that moved about at random (see Methods for additional details). Coherency here reflects a signal-noise ratio similar to that used in Random Dot Motion assays (RDM)^35,37,43^, but it also provides a visual correspondence to the changes expected in a fish’s visual field as the degree of organized motion in its group ranges from disorder (random motion with no leaders, *C *= 0) to order (perfectly aligned with only leaders, *C* = 1). In the null condition there are no leader silhouettes to provide directional cues (*C* = 0) and so the program randomly designates one arm over the other as the ‘correct’ choice to remain consistent with the remaining coherency levels in which leader silhouettes are present. With this in mind, the probability of making a correct decision by chance alone is a function of both the program and the zebrafish independently choosing the same arm (25%). When leader silhouettes are present $(C > 0)$ we controlled the relative strength of their directional cues by having the leader silhouettes either move at the same speed as the distractors, or ten times faster (Δ*v*:1, 10; see Movies S1 and S2 as examples). These Δ*v* values reflect general categories of fish swimming behavior, ranging from energetically sustainable speeds to costly burst of acceleration that are generally saved to avoid harm or capture prey^44–46^. In addition, this range of speeds has the potential to influence collective dynamics across a wide span of group sizes^11^. In the absence of leaders all visible silhouettes move at the same speed at the null condition, either slowly at the baseline speed (1 body length per second), or quickly (10 body lengths per second). In essence, by controlling the coherency and relative speed of the stimuli (leader silhouettes) we mimic the basic visual elements that social aggregations face in the wild, as individuals must make rapid directional decisions amidst varying levels of uncertainty and urgency. Our response variables are the speed and accuracy with which the fish then react to these visual stimuli. The time taken to traverse the decision zone defines the decision time, *T*_*D*_, while accuracy is a Boolean response indicating whether the fish has followed the leader silhouettes or not (see Methods).

### Individual effects

**Figure 2:**
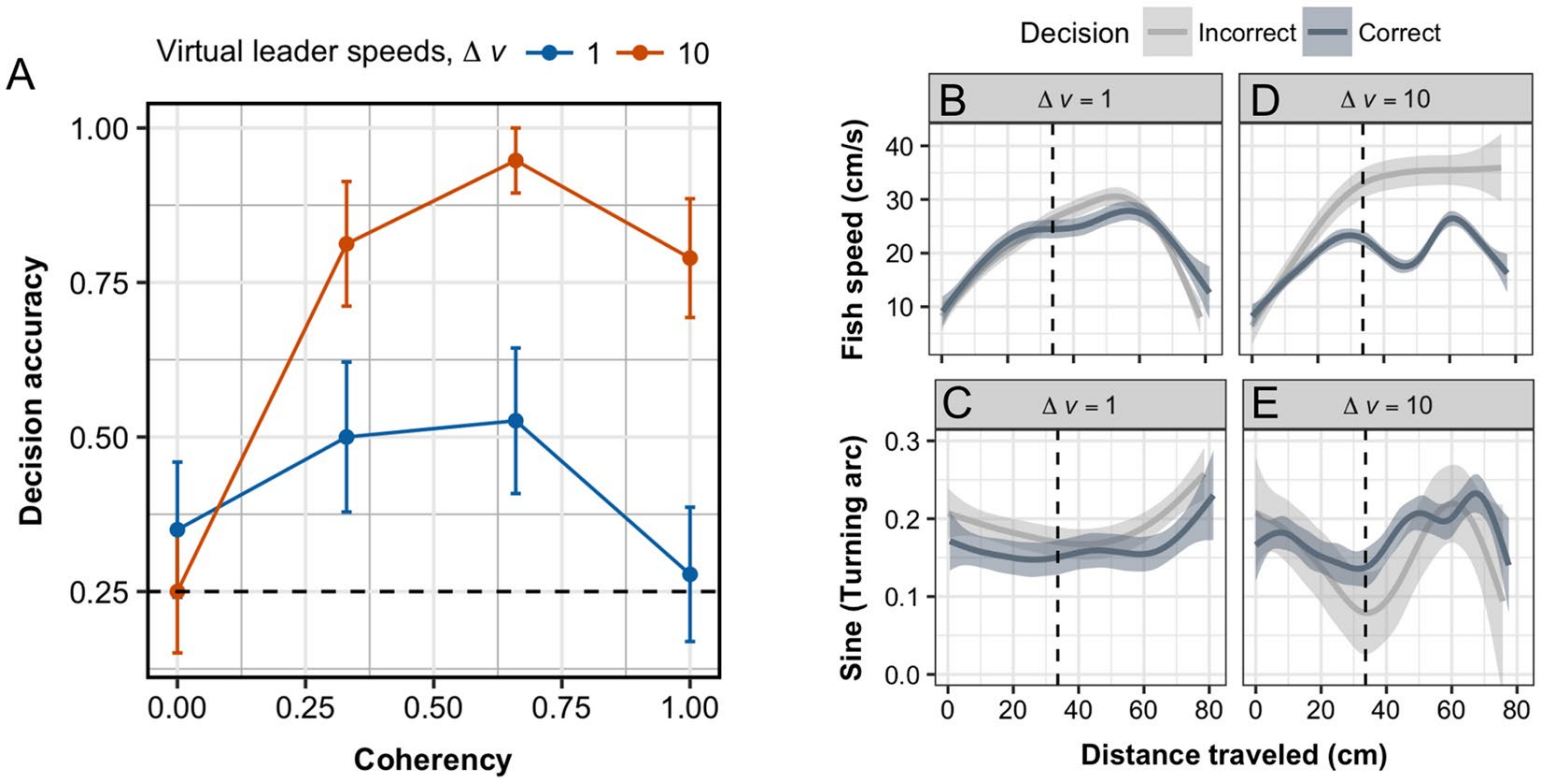
Decision accuracy and movement behaviors of solitary zebrafish. Mean decision accuracy (**A**) based on whether solitary zebrafish followed the visual stimuli as a function of the Coherency (*C*, x-axis) and relative speed (Δ*v*, color) of the projections. Data in (**A**) are mean ± Standard Errors (SE) and the horizontal dashed line indicates the null expectation (25%). Panels B–E show mean trends in the swimming speeds (**B,D**) and turning behaviors (**C,E**) of fish that either made the correct decision to follow the stimuli (dark gray) or not (light gray) and organized by the relative speed of the stimuli Δ*v* (columns). Data in panels (**B**–**E**) are pooled across all coherency levels in which directional cues were present, i.e., $C > 0$. Vertical dashed lines in (**B**–**E**) indicate the entrance of the decision zone. Turning arc measures the absolute angle between velocity vectors at time *t* and *t* − 1, ranging from 0 − *π*, and scaled using the sine function to range from 0−1. Data in (**B–E**) show mean trends 95% Confidence Intervals (CI).

Results of the solitary trials show that the coherency and speed of the visual cues had no effect on decision speed (Supplementary Table S1), but both factors had a significant interactive effect on decision accuracy (GLMM; *z* = 3.013, *P* = 0.003; Supplementary Table S2). Figure 2A shows how decision accuracy was equivalent between Δ*v* treatment levels when no motion cue was present (*C* = 0), but appears to be differentially influenced by Δ*v* as coherency increases. Under the null condition fish showed no directional bias for either arm (binomial test, *n*_left_ = 16, *n*_right_ = 24, $\hat{p}=0.40$, *P* = 0.268). When stimuli were present $(C > 0)$ fish were equally likely to choose either arm in the maze when Δ*v* = 1 (binomial test, *n*_correct_ = 24, *n*_incorrect_ = 31, $\hat{p}=0.44$, *P* = 0.419), but were nearly 6 times as likely to follow the stimuli than to miss them under the Δ*v* = 10 treatment (binomial test, *n*_correct_ = 46, *n*_incorrect_ = 8, $\hat{p}=0.85$, *P* = 0.002). We therefore conducted a series of post-hoc analyses to resolve the nature of the interaction and adjusted our *P*-values accordingly. A simple model without coherency confirmed that increases in speed significantly elevated decision accuracy (*z* = 3.337, *P* < 0.001). Modeling the effects of coherency on accuracy for each speed level separately revealed that coherency’s influence depended entirely on the relative speed values tested. When leader and distractor silhouettes moved at the same speed (Δ*v* = 1), changes in coherency had no effect (*z* = −0.364, *P* = 0.716; Fig. 2A, blue data points). In contrast, all coherency levels significantly influenced accuracy in the presence of the faster moving leaders (Δ*v* = 10, $C > 0$; Fig. 2A, red data points) and each of these were equally different from the control level (*C* = 0) (Supplementary Table S3). Effectively, while both speed and coherency initially appear to influence the fishes’ ability to follow the leader silhouettes $(C > 0)$, only the relative speed treatment results in a significant improvement in the average response.

To better understand the behavioral changes driving the observed decisions we analyzed the movement trajectories of our subjects. Panels B–E in Fig. 2 show swim velocities decomposed into their speed and turning components as a function of the cumulative distance traveled when directional stimuli were provided $(C > 0)$. Regardless of whether fish made the correct choice or not, those exposed to visual stimuli moving at the same speed as the distractors showed negligible differences in their swim speeds and turning behaviors, (Δ*v* = 1; Fig. 2B,C). In contrast, fish that correctly followed the faster moving stimuli (Δ*v* = 10) displayed a distinct deceleration upon approaching the decision zone (Fig. 2D). It is unclear whether individuals slowed down to inspect the projections beneath them or had a better chance of detecting them because they showed a tendency for prolonged exploratory behavior before committing to an arm. The results do, however, suggest that the average individual in these conditions displays a typical speed-accuracy trade-off (SAT), whereby decision accuracy increases with the time taken, or afforded, to make a choice. To resolve this question we compared the time individuals spent in the decision zone as a function of decision accuracy. We found that fish who correctly followed the leader silhouettes in the Δ*v* = 10 treatment took nearly twice as long to make a decision than their counterparts who chose incorrectly (2.6 ± 2 s *vs*. 1.3± 0.8 s, respectively; Wilcoxon rank sum test, W = 79, *P *= 0.01). In contrast, we found no evidence of an SAT in the Δ*v* = 1 condition (Supplementary Figure S2).

### Group size effects

While single zebrafish showed a strong optomotor response to faster moving stimuli, regardless of cue coherency, it remained unclear how social feedbacks from real shoalmates would modulate such reflexive, individual-level actions. To test the hypothesis that motion-guided attention can tune social interactions for groups on the move, we fixed the coherency of our visual stimuli, while manipulating Δ*v* and the number of live shoalmates introduced into the maze together. Fish were released into the maze in groups of 1, 5, 10, and 15. Coherency was fixed to the empirically derived level that maximized individual performance (*C* = 0.67; Fig. 2A) and stimulus speed treatment levels remained consistent (Δ*v*:1, 10). Fish were marked with a fluorescent elastomer injected beneath the skin at the base of their dorsal fin using different colors to facilitate recognition of focal subjects, whose decisions we recorded across treatments (See Methods for additional details and Movies S3 and S4 for examples). Here we also distinguished between the time taken for subjects to decide (i.e., traverse the decision zone, *T*_*D*_) and the time taken to act, *T*_*A*_, which is the time from which subjects were free to leave their holding area (gate up) until they made a decision to enter one of the opposing arms in the maze. The distinction is important since subjects were exposed to social stimuli while in the holding area, which is where social recruitment began. In the wild *T*_*A*_ would reflect the duration of an encounter for groups on the move, during which the average individual is more likely to react to the actions of their neighbors before they themselves have the opportunity to act on any information directly (e.g., decision time, *T*_*D*_).

**Figure 3:**
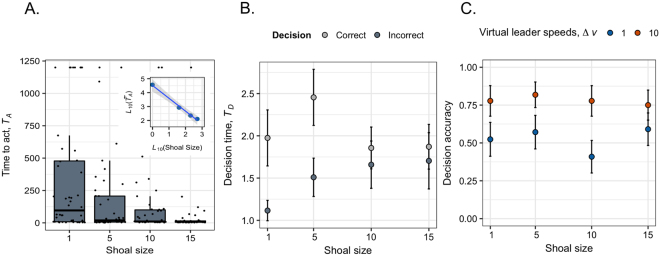
The effects of shoal size on the timing and accuracy of directional decisions. Boxplots showing the full range of time taken for subjects to leave the holding area to either make a decision or not (*T*_*A*_; A). Inset of (**A**) shows the median times taken on a log-log scale and does not obscure any outliers. Panel (B) shows the time taken to traverse the decision zone when selecting an arm (decision time, *T*_*D*_). Panel (C) shows the combined effects of shoal size and the relative speed of the visual cues on decision accuracy. Data in (**B**) and (**C**) are means ± SE.

Figure 3A shows how shoal size had a significant and pronounced effect on the average time taken for fish to act, with *T*_*A*_ decaying rapidly with increasing shoal size (LMM, *F*_1,152_ = 29.268, *P* < 0.001, Supplementary Table S4). Additionally, the median values of *T*_*A*_ decay as a power law of shoal size (${T}_{A}\sim {N}^{-0.923}$; *R*^2^ = 0.991; Fig. 3A inset). This relationship is germane because an exponential distribution is a reliable model for the likelihood of an event occurring and the median of this distribution should scale as a power law. Biologically, this trend infers that individuals will only need a few social interactions to show large increases in overall performance speed during events. Shoal size by itself, however, did not influence decision time (*T*_*D*_: *F*_1,137_ = 0.333, *P* = 0.565, Supplementary Table S5). As in the solitary trials, we found that those fish that moved more slowly through the decision area tended to make better decisions (GLMM, *z* = 2.075, *P* = 0.038); a pattern that is observed in groups of 1 and 5 fish but diminishes as group size increased further (Fig. 3B). Correlations between decision time and accuracy were weak, but positive, and decayed in strength with increasing group size (Pearson correlation coefficient per group size: *r*_1_ = 0.30, *r*_5_ = 0.28, *r*_10_ = 0.09, *r*_15_ = 0.06). In other words, subjects were increasingly likely to be recruited to act as neighbor presence grew, yet any boost in speed occurs before individuals have been exposed to the directional stimuli and, in fact, obscures the speed-accuracy trade-off operating at the individual level. Eventually a balance is struck between the time scale of the social recruitment process and individual decision-making as shoal size increases.

There was no effect of shoal size on subject decision accuracy (GLMM, *z* = −0.309, *P* = 0.757, Supplementary Table S6). However, the large difference in the relative speed of the visual stimuli significantly impacted decision accuracy regardless of shoal size, with fish consistently displaying a stronger tendency to follow the faster moving stimuli (Fig. 3C. *z* = 3.616, *P* < 0.001; Supplementary Table S6). We found no evidence of any interaction between shoal size and stimulus speed (Supplementary Table S6).

**Figure 4:**
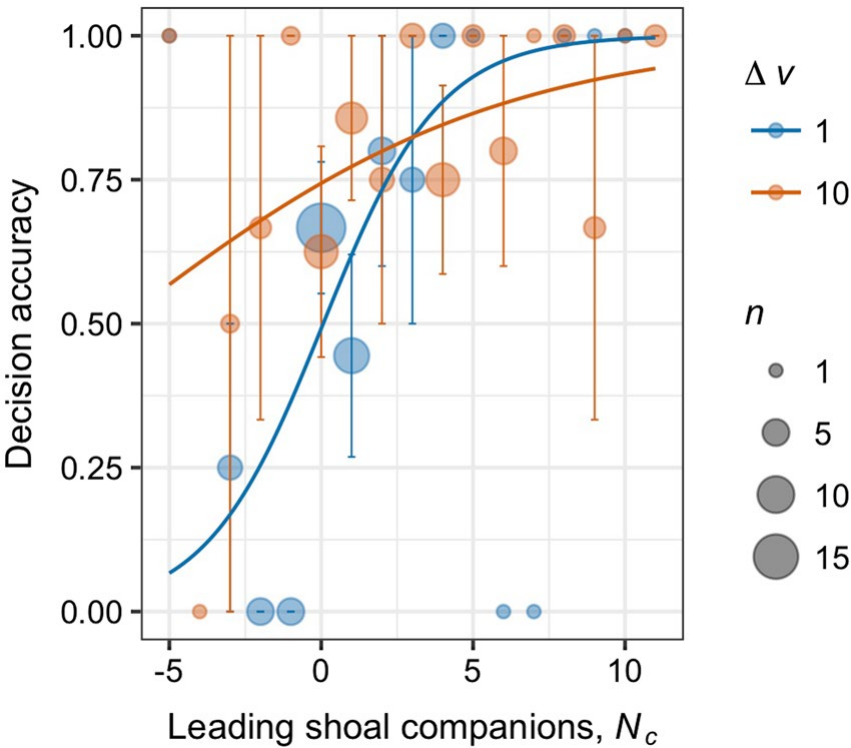
The effects of leading companions and the relative speed of the visual stimuli on decision accuracy. The proportion of accurate decisions are plotted as a function of the number of neighbors ahead of the subject who have already made a correct decision minus those who choose incorrectly, *N*_*c*_. Data are also grouped by the relative speed of the visual stimuli (Δ*v*; color). Data points are means ± SE and solid lines show the best fit of the logistic model within Δ*v* treatments (model parameters: Δ*v* = 1, a = 0.52, b = 0.07; Δ*v* = 10, a = 0.16, b = −6.73). Circle sizes reflect the number of observations for each *N*_*c*_ value and denoted by *n*.

The apparent lack of any social feedback in the decision process was initially surprising and, ultimately, misleading. The majority of subjects chose correctly (65%; binomial test: *n*_correct_ = 106, *n*_incorrect_ = 58, *P* < 0.001) even though the two speed treatments were evenly divided among them (binomial test, *n*_1_ = 86, *n*_10_ = 78, *P* = 0.585). This departure from our expectations likely stems from the dynamic nature of a shoaling life history strategy, whereby fish typically display a flexible collective structure rife with fission-fusion dynamics. Group structure varied within and across trials regardless of the initial group size, from tightly knit to fragmented. Yet, while individuals could be widely dispersed throughout the maze during a trial, subjects were found in the front half of the group more often than not (Supplementary Figure S3). Individuals in frontal positions often contribute more to collective movements than those behind them^18,47^, suggesting that our subjects were either in, or close to, positions of influence. To control for this spatial heterogeneity, test for social feedbacks, and better capture the subjects’ visual perspective, we tabulated the number of shoal companions that had already made a choice before the subject and recorded their decision. Following the approach by Ward *et al*. (2009)^9^ we then asked how subject decision accuracy changed as a function of the number of leading companions that choose correctly minus those that did not (*N*_*c*_). *N*_*c*_ therefore reflect whether the decisions made by companions prior to the subjects were either largely wrong (negative), split (zero), or correct (positive). In doing so we found that both the speed of the virtual leaders (GLMM, *z* = 2.909, *P* = 0.004) and the number of lead companions (*z* = 2.391, *P* = 0.017) significantly influenced subject decision accuracy (Fig. 4; Supplementary Table S7). There was no sign of any interaction. Accounting for the decisions of lead companions had no discernable effect on decision speed (Supplementary Table S8).

Figure 4 reveals that despite little indication of an interaction between Δ*v* and *N*_*c*_, the decisions of leading fish appear to influence individual choice differently depending on the relative strength of the visual cue, Δ*v*. To further evaluate this pattern statistically we parsed the data by Δ*v* and fit a logistic model to each group of Δ*v* data (1/(1 + *exp*(−*a*(*N*_*c*_ − *b*)))). The parameter controlling social feedback in this model, *a*, is significant when the cue is relatively weak (Δ*v* = 1) and matches the background level of virtual motion (Fig. 4, blue trend; *a* = 0.481, *t*_61_ = 2.355, *P* = 0.022). Under these conditions individuals were more susceptible to following the crowd (majority rule), whereas when fish were exposed to the faster moving cues (Δ*v* = 10) parameter *a* was not significant (red data; *a* = 0.161, *t*_59_ = 1.57, *P* = 0.122). The relative influence of parameter *a* across speed conditions is a trend that holds in each shoal size tested (Supplementary Table S9). Overall these data demonstrate that the speed of visual motion cues relative to the background level of motion (here the distractors) plays a significant role in modulating the influence that social interactions have on zebrafish directional decisions. When the visual cue is strong relative to the background level of motion, individuals are generally responsive regardless of neighbor activity, whereas when the cue is relatively weak individuals rely strongly on neighbor directional decisions to guide their way.

## Discussion

Our results have uncovered an interesting nuance to social behavior in a species displaying dynamic social ties. The speed and accuracy of decision making has been shown to increase with group size in shoaling fish when they are threatened with predation^9^, yet collective responses in social groups of varying cognitive complexity can be context- dependent^29,48,49^. When group members behave democratically, and integrate all sources of social information equally, the result is an increased resistance to error through averaging. In contrast, collective amplification relies on individuals reducing social interactions through some form of selectivity. Here we demonstrate how a biologically meaningful change in the speed of a primary visual feature, motion, plays a significant role in tuning social influence. Objects moving much faster than the background level of motion attracted the attention of fish and guided their directional decisions in groups. Individuals displayed robust directional responses to the faster motion cues regardless of shoal size, while local social interactions led to democratic decisions in the absence of any discernible motion cue and indirectly improved overall performance speed.

There is considerable evidence that social animals on the move may respond to their neighbors through a quorum rule, whereby individuals adopt the decisions of others once a threshold number of them have already committed to an action^50^. Quorum-like responses can be highly variable and difficult to replicate^29^, which suggests that they may either reflect the animal’s ability to tune their social response adaptively^50^ or mask a simpler reflexive mechanism^51^. Recent work also provided evidence that the spread of influential behaviors in schooling fishes relies not on the absolute number of stimuli, but their fraction in the observer’s field of view^13^. If so, a pertinent question is ‘what determines who is influential?’. The visual stimuli in our study system were projected from below onto a flat surface and were thus only expected to become apparent to the fish as they approached them, at which point the projections fell within their fields of binocular and lateral vision^31^. The maximum movement speeds of these virtual fish were also scaled to reflect those adopted by fish displaying a reflexive escape maneuver^46^ - thereby lending the cue adaptive significance. The fact that subjects in the first experiment consistently followed the faster moving virtual leaders, regardless of the coherency of these visual cues, provides strong evidence that it was the large difference in the speed of the cues and not their direction or relative numbers that drew the attention of individuals in the maze.

In groups, the speed of the visual stimuli continued to play a dominant role in guiding directional decisions regardless of group size. Data demonstrate that movement coordination within zebrafish shoals tends to peak at modest group sizes (*N* ≈ 5–20)^30^ and group size effects on decision making have been reported in similarly sized shoals of mosquitofish, *Gambusia holbrooki*^9^. Taken together with the fact that we found individuals to be unresponsive to changes in coherency levels within their visual field, it seems unlikely that the range of sizes tested here were insufficient to capture any group size effect on decision-making.

Interestingly, the differences we tested in the relative speed of the motion cues appear to have had little direct impact on the decision speeds of our subjects. Social interactions, however, were clearly expediting the speed at which members acted in a given situation (time to act, *T*_*A*_). Individually, members displayed the same speed-accuracy trade-offs generally expected in decision-making tasks, with better decisions being associated with longer decision times. As the time taken to act includes the individual’s decision time in our system, social interactions resulted in faster overall performance times by the average group member despite the underlying speed-accuracy trends operating on the individual. For instance, those subjects that followed the visual cue tended to take a second longer to decide than those that did not, whether they were tested alone or in groups of five (Fig. 3B), yet the median time taken for individuals to venture out and decide (*T*_*A*_) decreased by 81% when traveling in groups of five (Fig. 3A). That median *T*_*A*_ values decayed as a power law of shoal size reaffirms that this benefit needs only a few interactions to improve performance, which would be particularly advantageous during encounters for species living in fission-fusion societies where interactions can be ephemeral. Timing is everything, and the time taken to act is likely to trump accuracy when trying to survive the early stages of an attack, given that bursts of speed and variable movements are both important in avoiding predation^46,52^.

Social feedbacks through localized interactions did play an important, albeit supportive role, in determining individual directional choices, with changes in the visual cues’ speed influencing the recruitment process (Fig. 4). Animals often vary their speeds when traveling in groups, slowing down during turns, or adjusting speeds to maintain proximity. For animals traveling in groups, leadership roles often appear to stem from simple differences in speeds, which can significantly influence a group’s emergent behavior^22,53–55^. Faster moving individuals invariably find themselves at the front of traveling groups where they can have a disproportionate influence on the emergent direction^56^. However, simply displaying a sustained faster pace within a group does not necessarily reflect knowledge, or leadership, in and of itself^56^. For this reason, it is important that group members be able to adaptively tune their reliance on social information, so as to know when to follow their own instincts versus those of others in the group. Bursts of speed, while ephemeral, are energetically costly actions typically reserved for capturing prey and evading predators, thereby providing crucial roles in defining a mobile animal’s fitness that extend beyond notions of stamina. In the absence of such costly cues, an increased tendency to follow the actions of others would also be selectively advantageous by reducing the individual’s chances of wasting precious energy on false alarms, or becoming separated from the group in the face of uncertainty. What our analyses did not address is how individuals integrated the speed cues in their field of view or how intermediate values of Δ*v* influence the individual’s directional response. Would decision accuracy increase smoothly as Δ*v* is increased or would one find a sharp transition in behavior? Such information would provide valuable insight on whether individuals display selective attention to visual cues and if they adopt a threshold or a graded response to neighbor activity levels.

While vision has long been known to play an important role in fish shoaling behavior, we have demonstrated how a large difference in the relative speed of a visual cue can significantly influence directional decisions within groups and subsequently tune social interdependencies. Motion cues in an animal’s field-of-view are ecologically salient features that capture visual attention^57,58^ and bias directional decisions, thereby playing a critical role early on in the decision-making process by impacting how information flows through groups.

## Methods

### Subjects

Wildtype zebrafish were purchased from the University of Wisconsin, Milwaukee, kept under a day:night lighting cycle using flicker-free halogen lights, and fed Zeigler Zebrafish Diet (Zeigler Bros, Inc., Gardners, PA) daily.

### Experimental Domain

Experiments were conducted in accordance with federal and state regulations and approved by ERDC’s Institutional Animal Care and Use Committee (ERDC IACUC #3284-20143-1). The experimental domain is a watertight Y-maze set inside a larger 1.3 × 1.3 m elevated support platform surrounded by a privacy barrier. The arms of the maze within the domain are 46 × 23 × 20 cm (length, width, depth) with a central decision zone approximately 46 cm in diameter. A gate was controlled remotely with monofilament to release subjects from the holding area. Water quality within the domain was monitored throughout our study and remained steady across experiments (22 ± 0.3 °C, 97.4 ± 1.3% dissolved oxygen, and pH of 7.8 ± 0.1) We exchanged approximately 30% of the maze’s water with the primary water source supplying the holding tanks prior to the start of each day’s trials. Heaters and air-stones were kept in the maze overnight and during mid-afternoon breaks to homogenize and stabilize water temperatures and oxygen levels, but were removed prior to conducting trials and omitted during experiments.

### Virtual fish stimuli

The virtual fish stimuli were created using the Java-based Processing platform (version 3.0.1)^59^ and rendered to appear as the silhouettes of conspecifics swimming below our subjects. Silhouettes were of the same size and shape as our average test subject (3.5 cm body length, measured as fork length, FL) and moved at baseline speeds typical of normal swimming conditions (e.g., sustained swimming speeds, ≈1 body length per second, FL ⋅ s^−1 ^^60^). New silhouettes were placed randomly within the decision zone of the maze. Leader silhouettes moved directly towards a target arm chosen at random at the start of each trial, while distractors moved according to a correlated random walk (CRW). Turn angles in the CRW at each time interval were drawn from a wrapped Cauchy distribution centered on the silhouette’s current heading with a standard deviation of 30°. Images were refreshed at 60 frames-per-second, which is beyond the flicker-fusion frequency reported for zebrafish^61^. The arm chosen by the leader silhouettes varied randomly from trial-to-trial in all experiments (binomial test, *n*_*Left*_ = 78, *n*_*Right*_ = 71, $\hat{p}=0.52$, *P* = 0.632).

A total of 60 silhouettes were rendered in experiment 1 (solitary trials) to saturate the decision area with stimuli, thereby maximizing the chance that a fish would detect the visual cues. Boundary conditions were periodic and limited to the decision zone. Fish would slowly ‘vanish’ by changing to the background color at a rate proportional to their speed once they cross the boundary. After crossing the boundary, silhouettes would reappear at random within the decision area in the same state (leader or distractor) and retain their last known trajectory. Both the density of silhouettes and the leader-distractor ratio (Coherency, *C*) thus remained constant in the decision zone. See the Movies S1 through S4 for examples of zebrafish navigating the maze in each experiment.

In experiment 2 (social feedback effects) we extended the boundary of the leader silhouettes and reduced the overall number of silhouettes generated. The approximately unimodal relationship between the coherency of the silhouettes and fish response accuracy in experiment 1 (Fig. 2A of the main text) suggested a possible negative effect on movement decisions near the boundary at high coherency levels. As a precaution we first extended the boundary conditions of leader silhouettes back by 13.8 cm to reduce any negative influence generated by the disappearance of numerous stimuli at an arm entrance. Additionally, while our subjects cannot see the entire projection area due to their perspective, zebrafish have been shown to avoid shapes larger than themselves^62^, which would align with an inherent predator avoidance strategy. A large number of small images could appear as a single large shape (i.e., common fate). A control experiment was run to ensure that changing the number of projected silhouettes did not affect individual response under these conditions. Coherency levels were fixed at 0.67, speed to 1 FL ⋅ s^−1^, and the total number of silhouettes varied from 0–60. Reducing the number of stimuli had no discernible effects on either decision speed (*LMM*, *F*_1,141_ = 0.4005, *P* = 0.528) or accuracy (*GLMM*, *z* = −1.141, *P* = 0.2538).

### Visual recordings and lighting

Trials were recorded from above at 60 Frames Per Second (FPS) using a Sony FDR-AX100 Handycam camcorder (Sony, San Diego, CA). Fish movements in experiment 1 were tracked at 10 FPS using Zootracer (Microsoft Research, 2014)^63^. During experiment 1 the domain was illuminated from above with 4 adjustable LED lights (113.8 ± 11.1 lux). The fish silhouettes were projected onto a rear projection film adhered to the underside of the Y-maze using a short throw projector (Epson PowerLite 470 XGA 3LCD; Epson America, Inc.). The projector was offset from beneath the domain to reduce glare from its bulb and the image was corrected using the keystone settings. Offsetting the projector from beneath the domain only partially avoided the hot-spotting effect, however, which is a reflection of the projector’s bulb that creates an unwanted light gradient on the screen. To control for this effect we filtered the hotspot by creating a background image whose light gradient opposed the one created by the projector (Supplementary Fig. S1). The light level across the gradient was measured as pixel intensity using ImageJ^64^ and fit to a Gaussian decay function, which resulted in a 6-fold reduction in light variability across the floor of the domain by the projector (coefficient of variation, cv, in pixel intensity: pre-filter cv = 0.18, post-filter cv = 0.03).

Lighting conditions in experiment 2 were designed to detect the fluorescent elastomer tags used to identify individual subjects. The LED lights were replaced with 11 watt UV LED Blacklight PAR30 Spot-Flood Light Bulbs (screw-in) focused on the decision zone to maximize the amount of reflectance from the elastomer tags. The change in lighting conditions reduced the overall illumination in the domain. To compensate, the colors used to render the background and virtual fish were modified to retain the same Weber contrast levels found in experiment 1 (≈0.3). The same background correction procedure used in experiment 1 was adopted to retain a uniform background.

The changes in the projections and lighting between experiments 1 and 2 did not appear to impact the decisions of our subjects. A qualitative example of this can be seen by contrasting Figs 2A and 3C in the main text, where we find little change in the accuracy with which solitary fish followed the virtual leaders as a function of Δ*v* (*C* = 0.67 in 2A and shoal size of 1 in 3C). A statistical comparison of these specific conditions across experiments revealed that the accuracy with which fish followed the stimuli as a function of Δ*v* was found to be independent of experimental set up (${\chi }_{\mathrm{(0.05,1)}}^{2}=0.112$, *P* = 0.738).

### Fish tagging

When zebrafish were released in groups of varying size it was important to identify subjects and record their behaviors across treatments. While the sophistication and availability of automated tracking programs has steadily increased in the last decade^65,66^, there remain limitations that can constrain experimental conditions and lead to information loss^55,66,67^ and so it is common practice to tag individuals to ensure identification^12^. Here, all fish were tagged to control for handling effects, but using two different colors to distinguish subjects from their shoalmates (yellow and pink, respectively). Movie S4 provides an example of the stark visual difference observed between the VIE tags of the subject and shoalmates. During tagging fish were lightly anesthetized in an immersion bath using in 110 mg ⋅ L buffered tricaine methane sulfonate (MS222). Visible Implant Elastomer tags (VIE, 1 ml elastomer: 0.1 ml curing agent) were injected using an insulin syringe (NMT 2006). Elastomer tags were injected along the dorsal fin base, 4 mm in length. After recovery from anesthesia, fish were moved to 10 L tanks in an aerated flow-through water system (≈0.4 L ⋅ min^−1^). Fish were given 10 days post VIE injection to recover and were afterwards visually inspected 3 times a day for tag loss and health status.

### Experimental Design and Statistics

#### Experiment 1

We housed 20 zebrafish subjects in individual tanks (2 × 8 × 18 cm). Each tank was divided in half by a transparent barrier that separated the test subject from a group of conspecifics, which improved subject retrieval during trials and provided social stimuli (approximately 3–4 conspecifics) to reduce any potential stress from isolation through social buffering^68^ – an effect that has been subsequently demonstrated in zebrafish^69^. We varied the proportion of virtual silhouettes that were leaders using 4 coherency levels ranging from no leaders to only leaders (*C*: 0.0, 0.33, 0.66, 1.0). When there are fewer leaders than distractors amongst the silhouettes (*C* < 0.5), the projection is dominated by disorganized motion and the directional cue is weak. Correspondingly, when all silhouettes are leaders, the directional cue is unambiguous (*C* = 1). We also varied the speed of the virtual leaders to either move at the same speed as the distractors, or 10 × faster (Δ*v*: 1, 10). For example, at *C* = 0.33 and Δ*v* = 10 a third of the silhouettes moved directly towards a randomly selected arm at a speed of 35 cm ⋅ s^−1^ (≈10 FL ⋅ s), while the remaining silhouettes moved randomly at 3.5 cm ⋅ s^−1^ (≈1 FL ⋅ s).

Trials were organized in a full factorial, repeated measures design with all 20 subjects exposed to all *C* × Δ*v* combinations. Trials took place between 9 a.m. and 3 p.m. We used a Linear Mixed Model (*LMM*) to test if the relative speed and coherency of the stimuli affected the decision time of our subjects (time values were transformed using the natural logarithm). Fish id was included as the random effect to account for the repeated measures. A Generalized Linear Mixed-Model (*GLMM*) with binomial error was used to test whether the coherency and relative speed of the virtual silhouettes affected decision accuracy. We found no evidence of any directional bias in our subjects, in terms of the arm selected in the maze, that could lead to spurious results in our models (binomial test on the probability turning Left or Right; *n*_*Left*_ = 65, *n*_*Right*_ = 84, $\hat{p}=0.44$, *P* = 0.14). All data processing and statistical analyses were done in R (version 3.0.2).

We quantified swimming behavior in the maze using a kinematic analysis of the positional data collected with the tracking program. Positions were recorded at 12 FPS and smoothed using a running median with a 3-step window. Velocity vectors were then estimated from numerical differentiation of the smoothed positions over time, normalized to mean body length, and scaled to seconds (FL ⋅ s^−1^). Accelerations were then calculated by differencing the velocities as a function of time. Turning behavior was quantified by differencing vector orientations from one time step to the next. Subjects rarely made turns in excess of 90° (<2% of all observations) and so we applied a sine transformation to normalize turning arcs to a dimensionless quantity between 0–1.

#### Experiment 2

A total of 24 zebrafish subjects (mean FL = 3.77 ± 0.39 cm) were exposed to two factors, the relative speed of the virtual leaders (Δ*v*) and fish shoal size (e.g., the number of real fish introduced into the maze with the subjects to create the shoal). We tested the same two speed levels used in Experiment 2 (1, 10) and tested four group sizes (1, 5, 10, 15). In accordance with our institutional animal care protocols, we reduced the number of subjects necessary by using a main population of test subjects, supplemented by a companion population (*N* = 162) to serve as social companions to supplement each group level, i.e., shoals of $N > 1$ were composed of a subject and *N* − 1 companion fish). Test subjects were exposed to each factor combination in a repeated measures, semi-systematic design. Companion fish were housed in an entirely separate population from the subjects and divided equally into three sub-groups assigned to specific trial days (54 fish ⋅ tank^−1^). We blocked our design by population and factor to reduce the risk of carry over effects and stabilize the number of fish used per day. Trial sequences were as follows. We conducted 8 trials per day, with 3 days on and 1 day off over the span of 4 weeks. Subject fish were tested in ascending sequence (by id number) in each 3-day block and each day had an assigned sub-population of companion fish; ensuring that all fish had 3 days off between trials. The 8 subjects of a given day’s trials were then randomly divided among each group size (2 fish ⋅ group size^−1^) to fix the number of fish used per day, and then randomly assigning a speed level. Trial sequences within each day were then randomized. Statistical procedures followed that of Experiment 1 to analyze the effects of each factor on our time variables (*LMM* using the natural logarithm of *T*_*A*_ and *T*_*D*_) decision accuracy (GLMM) with fish id as the random effect.

Given the fluid nature of shoaling behavior we repeated our analysis of decision accuracy by replacing overall shoal size by the number of lead companion fish, *N*_*c*_, that made their choice prior to the subjects. A binomial GLMM with fish id as the random effect was again employed. A total of 5 outliers drawn from both positive and negative *N*_*c*_ values were removed from the final model to correct for rank deficiencies that arose from only one observation per *N*_*c*_ level.

### Data availability

Data can be found at: https://github.com/brilraven/ZebrafishMotionCues and at the Knowledge Network for Biocomplexity (https://knb.ecoinformatics.org/#view/doi:10.5063/F1P26W9D).

## Electronic supplementary material

Supplementary Information

Movie S1

Movie S2

Movie S3

Movie S4
