# Supplementary material for: Motion cues tune social influence in shoaling fish
Source: Sci Rep. 2018 Jun 28;8:9785. doi: 10.1038/s41598-018-27807-1 (PMC6023868; doi:10.1038/s41598-018-27807-1)
Supplement: Supplementary file 1 — Supplementary Information [file 41598_2018_27807_MOESM1_ESM.pdf]

Supplementary Information:

Motion cues tune social influence in shoaling fish

Lemasson, B.H. [1†], Tanner, C.J. [2], Woodley, C.[3], Threadgill, T.L. [3], Qarqish, S.[3,4], and Smith, D.L. [3]

1. Environmental Laboratory, U.S. Army Engineer Research & Development Center, Newport, OR, USA
2. Misericordia University, Dallas, PA, U.S.A.
3. Environmental Laboratory, ERDC, Vicksburg, MS, U.S.A.
4. Current address: William Carey University, College of Osteopathic Medicine, MS., U.S.A

Contents

1 Figures 2

2 Statistical tables 5

[†]Author for correspondence: brilraven@gmail.com, bertrand.h.lemasson@usace.army.mil

1 Figures

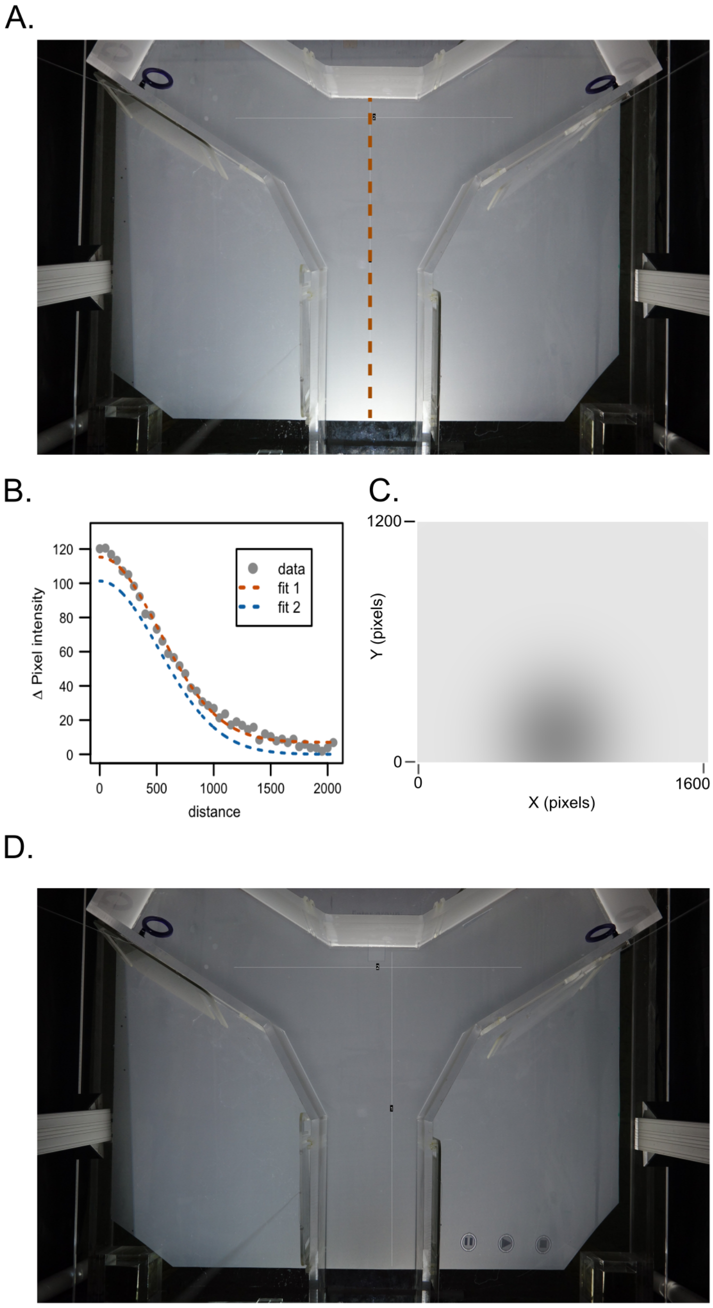

Figure S1: Calibration of the background projection. The projector's proximity and relative angle to the experimental domain created a light gradient (or hotspot) on an otherwise uniform gray background (**A**). Pixel intensity along a transect from the brightest location to the dimmest one (red dashed line, **A**) closely followed a gaussian decay (gray circles, **B**), which was fit with the function $a*e^{(-(x^2/(2*b^2)))}+c$ (fit 1, dashed red line, **B**). The best fit model was adjusted to eliminate any difference in pixel intensity across the gradient (fit 2, dashed blue line, **B**) and create an inverted background image (**C**) that substantially improved background uniformity (**D**). Model parameters: (fit 1) a = 108.3, b = 518.2, c = 7.0; (fit 2) a = 101.3, b = 518.2, c = 0.0).

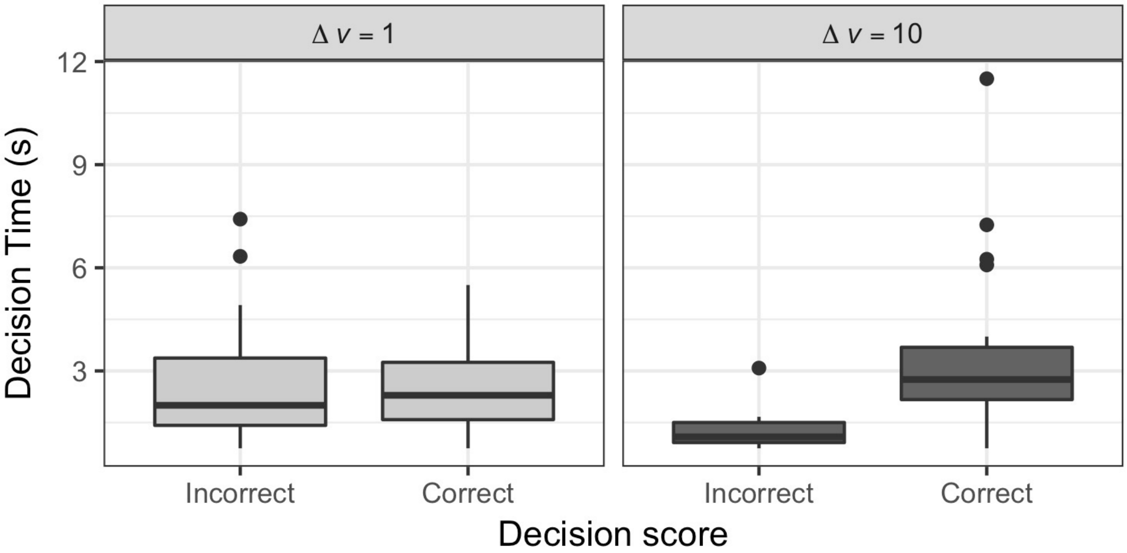

Figure S2: Decision time in the solitary zebrafish trials as a function of Δv treatment and decision accuracy. In the $\Delta v = 1$ treatment we find little difference in the time taken to traverse the decision zone based on whether fish correctly or incorrectly followed the leader silhouettes (taking 2.3 ± 1.6 s *vs.* 2.2 ± 1.4 s, respectively). In contrast, fish in the $\Delta v = 10$ treatment that correctly followed the leader silhouettes took significantly longer to cross the decision zone than their counterparts (2.6 ± 2.0 s *vs.* 1.3 ± 0.77 s, respectively; Wilcoxon rank sum test, $W = 79,\ P = 0.01$). Data include only trials in which leader silhouettes were present ($C > 0$) and are subset by Δv ($\pm$ here reflect standard deviations).

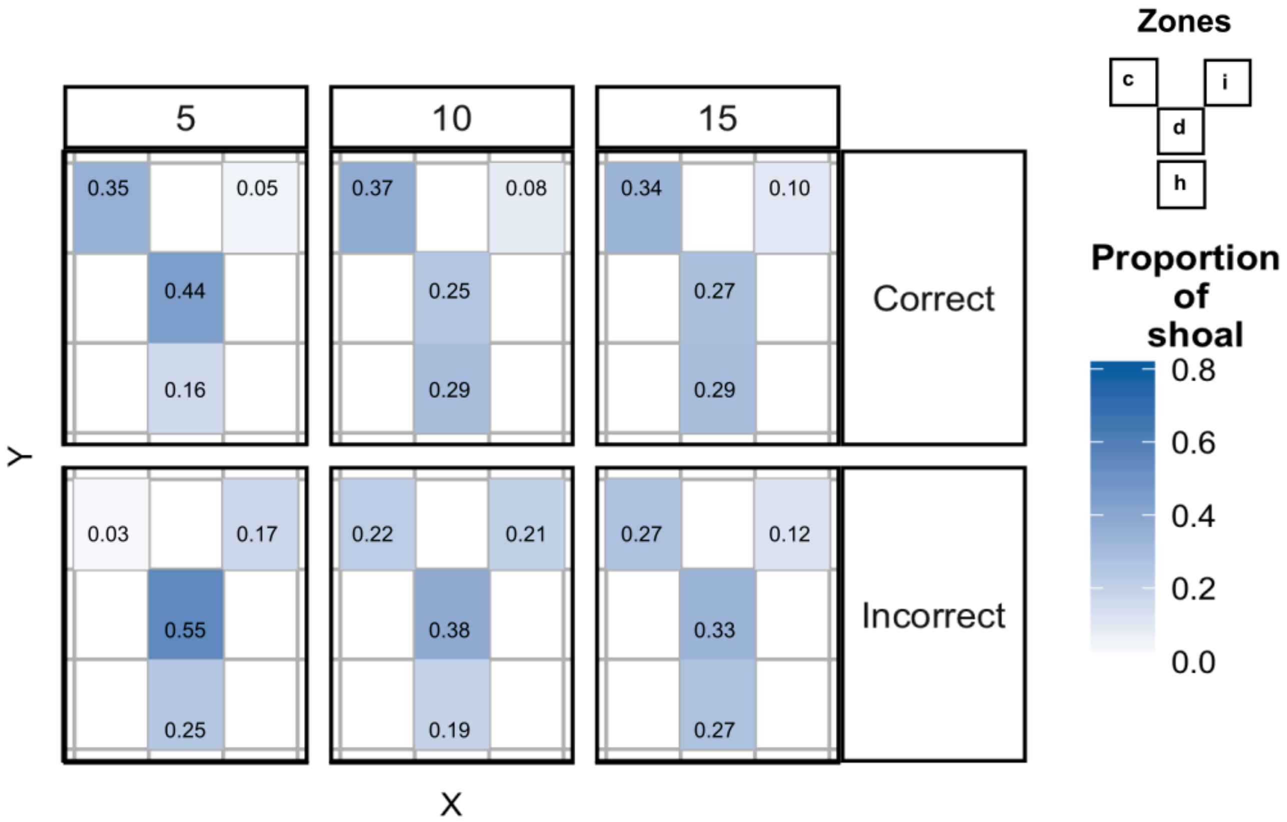

Figure S3: Spatial distribution of zebrafish in the maze when subjects made their directional decision. Panels show the proportion of fish found in different zones of the maze as a function of shoal size and whether or not the subjects followed the leader silhouettes into an arm. Fish that chose to follow the virtual leaders were deemed to have made a correct choice. Zones in each panel depiction of the maze correspond to the holding area (h), decision zone (d), correct choice (c), and incorrect choice (i; illustrated in the legend). Although the arm chosen by the virtual fish 'leaders' in each trial was selected at random, the positions of correct *vs.* incorrect arms are fixed here for visual simplification.

2 Statistical tables

Table S1 Linear Mixed-Model (LMM) results for experiment 1: Decision time, $\ln(T_D)$, as function of the relative speed of the leader silhouettes with respect to the distractors, Δv, and the overall coherency of the visual stimuli, C. Significance was determined in a stepwise fashion and results are presented for each step. DF is the ratio of degrees of freedom.

Fixed-effects	Step	DF	F	P
Δv x C	1	1/126	0.5374	0.4649
Δv	2	1/127	0.0291	0.8649
C	3	1/128	0.8152	0.3683

Table S2 Main GLMM results for experiment 1: Decision accuracy as a function of Δv and C.

Fixed-effects	Wald Z	P
Δv	-0.350	0.7267
C	-0.784	0.4332
Δv x C	**3.013**	**0.0026**

Table S3 Tukey's Honestly Significant Difference (HSD) post-hoc test on the reduced GLMM model of decision accuracy as a function of C for $\Delta v = 10$ data. Coherency is treated as a factor for this analysis to determine the effects of each level on the response variable. Coherency levels are given in brackets and all P values are adjusted using the Bonferroni-Holmes correction.

Coherency levels	z	P
0.00 – 0.33	**3.118**	**0.0037**
0.00 – 0.66	**3.469**	**0.0031**
0.00 – 1.00	**3.169**	**0.0037**
0.33 – 0.66	1.176	0.2874
0.33 – 1.00	-0.170	0.8653
0.66 – 1.00	-1.339	0.2708

Table S4 LMM results for Experiment 2: Time to act, $\ln(T_A)$, as a function of shoal size and the relative speed of the visual stimuli. A small percentage of subjects never committed to entering an arm (7.3% of all observations, $N = 13$ fish) and the majority of these fish were alone (8/13). Including all subjects in the analysis is ecologically relevant because undecided individuals represent situations where some group members may hesitate to act, which can have severe fitness consequences. However, to ensure that these indecisive fish were not biasing our results we repeated the analysis with decisive fish only and found no differences in our conclusions.

All fish

Fixed-effects	Step	DF	F	P
Δv x Shoal size	1	1/150	0.6010	0.4394
Δv	2	1/151	0.5298	0.4678
Shoal size	3	1/152	**29.26769**	**<0.0001**

Decisive fish

Fixed-effects	Step	DF	F	P
Δv x Shoal size	1	1/137	0.7246	0.3961
Δv	2	1/138	0.0439	0.8344
Shoal size	3	1/139	**18.5437**	**<0.0001**

Table S5 LMM results for Experiment 2:Decision time, $\ln(T_D)$, as a function of shoal size and Δ v.

Fixed-effects	Step	DF	F	P
Δv x Shoal size	1	1/135	0.8876	0.3478
Δv	2	1/136	0.35411	0.5528
Shoal size	3	1/137	0.33284	0.5649

Table S6 GLMM results for Experiment 2: Decision accuracy as a function of shoal size and $\Delta\ v$.

Fixed-effects	Step	Wald (Z)	P
$\Delta v\ x$ Shoal size	1	-0.238	0.8121
Shoal size	2	-0.309	0.7573
Δv	3	**3.616**	**0.0003**

Table S7 GLMM results for Experiment 2: Decision accuracy as a function of $\Delta\ v$ and the number of leading companions, N_c.

Fixed-effects	Step	Wald (Z)	P
$\Delta v\ x\ \ N_c$	1	-1.146	0.2518
Δv	2	**2.909**	**0.0036**
N_c	2	**2.391**	**0.0168**

Table S8 GLMM results for Experiment 2: Decision time, $\ln(T_D)$, as a function of $\Delta\ v$ and the number of leading companions, N_c.

Fixed-effects	Step	F	P
$\Delta v\ x\ \ N_c$	1	0.0936	0.7602
Δv	2	0.1816	0.6707
N_c	2	1.4954	0.2235

Table S9 Best fit parameters for the logistic models to estimate the strength with which leading companions, N_c, influenced the directional accuracy of the subject fish. Parameter values were estimate using R's nlsLM function in the minpack.lm package

Group data	Δv	N	a	SE	t	P
all (pooled across groups)	1	61	0.481	0.204	2.355	**0.022**
all	10	59	0.161	0.103	1.568	0.122
5	1	21	1.053	0.695	1.514	0.147
5	10	22	0.462	0.332	1.385	0.181
10	1	21	0.222	0.191	1.163	0.259
10	10	18	0.070	0.146	0.476	0.640
15	1	19	0.142	0.194	0.734	0.473
15	10	19	0.138	0.135	1.018	0.323
